# The Anatomy of the Horse

**Published:** 1885-04

**Authors:** 


### REVIEWS.

The Anatomy of the Horse. A dissection guide by Prof. McFadyean, M.B., C.M., B.Sc. Published by Wm. R. Jenkins, Veterinary Publisher and Bookseller, 850 Sixth Avenue, New York. [All rights reserved.] Pp. 374.

This is the first volume of a veterinary character, appearing in the English language, that we have been able to lay down with unfeigned and unmixed satisfaction. Certainly nothing could be more thorough, and expressed in more intelligible language, nor anything in anatomy illustrated better than has been done in this work. The arrangement and classification of the subject matter is beyond criticism. The author has not contented himself with an enumeration and description of anatomical features, aided by figure references, but leads his readers right up to the dissected animal, so minute in all his directions, so reliable in every statement of detail, that with this book and a case of instruments, unaided by any teacher, a student could become an expert anatomist and dissector.

In accordance with the general plan of his work, Doctor McFadyean omits all introductory remarks, and plunges *in medias res*, beginning with the dissection of the pectoral region. In the study of each anatomical locality he states, first, in what position the limb is to be dissected, and then furnishes what is a desideratum in all other veterinary dissecting guides, and attempted, to the best of our knowledge, only in "artists' dissections," a surface topography, indicating the outer markings of the horse's body and their relations to the parts beneath.

We are unable to point out a single error of importance, and even in that chapter where the author alone exhibits some doubt or uncertainty, we see evidences that he has everywhere based his descriptions and figures on his own dissections, and not, as is too often done, and notably so in the case of several recent manuals, on abstracts from Chauveau, Leyh, and others. We have been struck with the fact that hitherto the anatomy of the nervous system of the horse—probably the most neglected, because the most important, next to that of the foot and intestine—has been based on the anatomy of the human brain. Unfortunately, Hippo-anatomists have taken as their models not the best, but the worst descriptions of the human brain. The consequence is that in such a work as Chauveau, for example, the thalamus is enumerated as contained in the lateral ventricle. It is now well settled that in all animals, the greater mass of the thalamus is extra-ventricular. McFadyean shows that he is a cautious writer, and does not speak of things unseen by him, in the foot-note on page 249: "The optic thalamus and taenia semi-circularis are generally enumerated among the objects visible in the lateral ventricle. In the brain of the horse, however, the choroid plexus completely covered from view the optic thalamus and in most cases the taenia semi-circularis." When the student tears away the choroid plexus he destroys the partition which bounds the lateral ventricle internally, and which is attached parallel to the taenia in the innermost one-twentieth of the thalamus (Ripa of Wilder). Among the omissions of the index in this connection, is the "Fimbria," which, in one of the figures, is named "taenia hippocampi."

The illustrations in the text are ample, and forty-eight magnificent colored plates from the author's own dissections, true, for the most part, to nature, are scattered through the volume. We are unable to understand how the publishers are able to offer the work at so low a figure. We have no other suggestions to make with regard to a second edition, which we have no doubt will be called for, than the addition of a plate illustrating the relations of the floating and fixed color, and the substitution of better plates for those showing the brain. The drawings made by the author are entirely true to nature and reliable, but those two, drawn by a professional artist, are below the high standard of the remainder.

In conclusion we can only congratulate the student of anatomy, that a want so long and so grievously felt is at length supplied, the author in having done his work so well, and the publisher for his enterprise and liberality in the line of costly illustrations. E. C. S.

